# Effects of probiotic supplementation on markers of skeletal muscle damage, perceived recovery and athletic performance after an intense single leg training bout

**DOI:** 10.1186/1550-2783-12-S1-P36

**Published:** 2015-09-21

**Authors:** Ralf Jäger, Kevin Shields, Matthew Sharp, Jeremey Partl, Jacob M Wilson, Ryan P Lowery, Eduardo O De Souza, Chase Holmer, Martin Purpura

**Affiliations:** 1 Increnovo LLC, 2138 E Lafayette Pl, Milwaukee, WI 53202, USA; 2 Department of Health Sciences and Human Performance, The University of Tampa, 401 W. Kennedy Blvd., Tampa, FL 33606, USA

## Introduction

The probiotic GanedenBC^30 ^(*Bacillus coagulans *GBI-30, 6086; Ganeden Biotech Inc., Maryfield Heights, OH) has been shown to support healthy digestive and immune function, including increased protein absorption. In a pilot study, daily co-administration of GanedenBC^30 ^and protein in resistance-trained subjects performing full body workouts 4 times per week for 8 weeks has shown a trend to increase vertical jump power and might have a beneficial effect on peak power and fat mass. We speculate that the beneficial effects might be based on aiding muscle recovery through gut microbial modulation. Thus, the purpose of this investigation was to determine if the co-administration of GanedenBC^30 ^with protein has a beneficial effect on muscle damage, recovery and athletic performance following a damaging exercise bout.

## Methods

30 healthy recreationally-trained males participated in this study (mean+/-SD; age: 21.5 ± 2.8 years; height: 177.4 ± 8.0 cm; weight: 89.7 ± 28.2 kg). Subjects were randomly assigned to consume either 20 g of casein (Control = CON) or 20 g of casein plus probiotic (500M CFU GanedenBC^30^, = BC30) twice daily in a crossover, diet-controlled design for a two-week time period. Subjects performed a damaging exercise bout consisting of 10 sets × 10 repetitions unilateral leg press at 70% 1 RM with 1 minute rest, one legged - leg extension (5 sets × 12 reps), and rear foot elevated split squat 5 sets × 12 reps with one minute rest at baseline and after two weeks of supplementation. Athletic performance consisting of peak power (Wingate 10 sec Peak Power Assessment at 7.5% BW at 175RPM threshold loaded drop), vertical jump power (Tendo unit, single-leg jump), and 1-RM single-leg press; and muscle damage was analyzed by muscle swelling (ultrasonography) and blood draws (creatine kinase (CK), blood urea nitrogen (BUN)) were taken at baseline (pre-supplementation) and 48 hours after damaging exercise bout. Perceptual measures (perceived recovery, soreness) were taken before, 24, 48 and 72 hours after exercise.

## Results

The damaging exercise bout significantly increased muscle soreness (p < 0.001), reduced perceived recovery (p < 0.001), however, BC30 significantly increased recovery at 24 and 72 hours, and decreased soreness at 72 hours post exercise in comparison to CON. Perceptual measures were confirmed by increases in CK (CON: +266.8%, p = 0.0002; BC30: +137.7%, p = 0.01), with BC30 showing a trend towards reduced indices of muscle damage (p = 0.08). The strenuous exercise significantly reduced athletic performance in CON (Wingate Peak Power; CON: (-39.8 watts, - 5.3%, p = 0.03)), whereas BC30 maintained performance by (+10.1 watts, +1.7%). There were no differences between groups for strength responses (CON: +7.2 kg, +2.6%, p = 0.15; and BC30: +3.4 kg, +1.2%, p = 0.79).

## Conclusions

This study indicated that probiotic supplementation in form of GanedenBC^30 ^in combination with protein (casein) reduces indices of muscle damage, increases recovery and may maintain athletic performance after muscle damaging exercise.

